# Apoptotic effect of novel Schiff Based CdCl_2_(C_14_H_21_N_3_O_2_) complex is mediated via activation of the mitochondrial pathway in colon cancer cells

**DOI:** 10.1038/srep09097

**Published:** 2015-03-13

**Authors:** Maryam Hajrezaie, Mohammadjavad Paydar, Chung Yeng Looi, Soheil Zorofchian Moghadamtousi, Pouya Hassandarvish, Muhammad Saleh Salga, Hamed Karimian, Keivan Shams, Maryam Zahedifard, Nazia Abdul Majid, Hapipah Mohd Ali, Mahmood Ameen Abdulla

**Affiliations:** 1 Department of Biomedical Science, Faculty of Medicine, University of Malaya, 50603 Kuala Lumpur, Malaysia; 2 Institute of Biological Science, Faculty of Science, University of Malaya, 50603 Kuala Lumpur, Malaysia; 3 Department of Pharmacology, Faculty of Medicine, University of Malaya, 50603 Kuala Lumpur, Malaysia; 4 Department of Chemistry, University of Malaya, 50603 Kuala Lumpur, Malaysia

## Abstract

The development of metal-based agents has had a tremendous role in the present progress in cancer chemotherapy. One well-known example of metal-based agents is Schiff based metal complexes, which hold great promise for cancer therapy. Based on the potential of Schiff based complexes for the induction of apoptosis, this study aimed to examine the cytotoxic and apoptotic activity of a CdCl_2_(C_14_H_21_N_3_O_2_) complex on HT-29 cells. The complex exerted a potent suppressive effect on HT-29 cells with an IC_50_ value of 2.57 ± 0.39 after 72 h of treatment. The collapse of the mitochondrial membrane potential and the elevated release of cytochrome *c* from the mitochondria to the cytosol indicate the involvement of the intrinsic pathway in the induction of apoptosis. The role of the mitochondria-dependent apoptotic pathway was further proved by the significant activation of the initiator caspase-9 and the executioner caspases-3 and -7. In addition, the activation of caspase-8, which is associated with the suppression of NF-κB translocation to the nucleus, also revealed the involvement of the extrinsic pathway in the induced apoptosis. The results suggest that the CdCl_2_(C_14_H_21_N_3_O_2_) complex is able to induce the apoptosis of colon cancer cells and is a potential candidate for future cancer studies.

Cancer, as the second leading cause of mortality worldwide, is a major health problem of global concern. Colorectal cancer is one of the most malignant neoplasia and is considered to be one of the three most prevalent types of cancer in both men and women^1^,^2^. The process of cancer development involves multiple steps in the initiation phase that make normal cells able to turn into tumours and lead to the promotion stage, which results in malignant growth and invasion in the progression stage^3^. In colorectal carcinomas, the transformation of normal colonic epithelium into carcinoma via the intermediation of adenoma is known as the adenoma-carcinoma sequence. Colorectal tumorigenesis arises from genetic and epigenetic alterations and the concurrent accumulation of histological changes. The accumulation of these perturbations, which are mostly related to the regulation and expression of the prominent genes of PIK3CA, PTEN, BRAF, c-myc, p53, APC, and K-ras and DNA mismatch repair genes, promotes the clonal expansion of tumour cells^4^,^5^.

Despite the remarkable achievements in precautionary measures and diagnosis techniques and the improvements in chemotherapy, the median overall survival period of colorectal cancer patients with metastatic is only 24 months. Moreover, chemotherapeutic agents should ideally only affect tumour cells, but the majority of the anticancer agents that are currently being used in the clinic exhibit numerous side effects on the human body, namely diarrhea, bleeding, hair loss, and immunosuppression^6^. Furthermore, the important clinical issue of resistance to current chemotherapeutic drugs also represents a critical challenge in cancer therapy and even after surgical resection; moreover, adjuvant therapy is still required for patients with colorectal cancer^7^,^8^. The growing body of molecular and experimental studies supports the crucial role of the evasion of apoptosis in the drug resistance and molecular pathways of carcinogenesis^9^.

The extensive application of metal complexes in the clinic for centuries presents a promising window that can be exploited for the discovery of potential therapeutic drugs, although the molecular mechanism underlying their biological activities has not yet been completely explained^10^,^11^. The development of metal-based drugs is deeply indebted to platinum-based antitumor agents, mainly including oxaliplatin, carboplatin and cisplatin, for the great successes achieved in cancer therapy. However, severe adverse side effects are associated with all of these platinum-based drugs^12^. In the last few decades, Schiff bases and their complexes have attracted significant attention in the field of coordination chemistry and have become well known for their extensive biological potential^13^. Condensation between carbonyl compounds and amines in different reaction conditions has been realized, and in different solvents. The formations of ketones derived Schiff bases (ketimines) have been successful in the presence of dehydrating agents. Acid salts (usually MgSO_4_ or Na_2_SO_4_) are commonly employed as dehydrating agents. Primary alcohols such as ethanol have been widely used as a solvent for the preparation of Schiff bases^14^. They have been purified by crystallization methods because separation of Schiff bases using silica gel can cause some degree of decomposition, through hydrolysis. If the compounds are insoluble in hexane or cyclohexane, they can be purified by stirring the crude reaction in a mixture of solvents, sometimes adding a small portion of relatively polar solvent e.g diethyl ether and dichloromethane, in order to eliminate impurities. In general, Schiff bases are stable solids and can be stored without many precautions^14^. A variety of biological activities, including anti-HIV, anti-fungal, anti-bacterial, herbicidal, antitubercular, and anticancer activities, have been elicited from Schiff metal complexes^15^,^16^. Numerous studies on Schiff bases with metal complexes of manganese, nickel, zinc, copper, and cobalt have been reported, although detailed scientific scrutiny of CdCl_2_(C_14_H_21_N_3_O_2_) complexes with Schiff bases and their biological activities is still required^17^. In the present work, we investigated the cytotoxic effects of CdCl_2_(C_14_H_21_N_3_O_2_) complex against HT-29 human colon adenocarcinoma cancer cells. In addition, we also examined the potential of this complex for the induction of apoptosis and suggested a possible molecular mechanism.

## Results

### The cytotoxicity effect of CdCl_2_(C_14_H_21_N_3_O_2_) complex on HT-29 and CCD841 cell lines

The results from the triplicate MTT assays on the HT-29 and CCD 841 cell lines demonstrated that the CdCl_2_(C_14_H_21_N_3_O_2_) complex does not exhibit any cytotoxicity on normal cells and does show a significant inhibitory effect on HT-29 cells. As shown in Table 1, the CdCl_2_(C_14_H_21_N_3_O_2_) complex elicited an IC_50_ of 2.57 μg/mL against HT-29 cells after 72 h.

### Cytotoxic effects of CdCl_2_(C_14_H_21_N_3_O_2_) complex by LDH release assay

**Figure 1:**
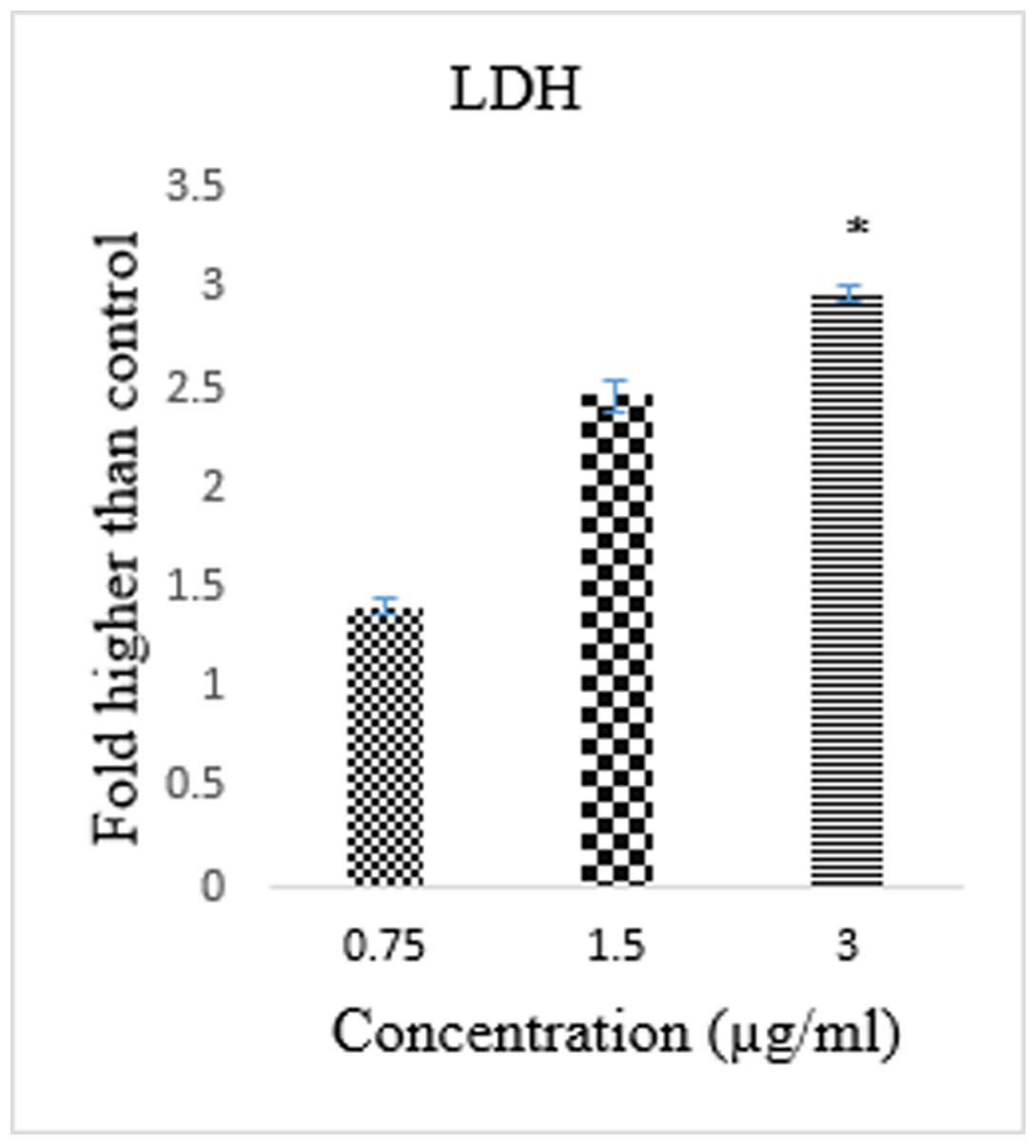
Lactate dehydrogenase (LDH) assay, demonstrated the cytotoxicity of CdCl_2_(C_14_H_21_N_3_O_2_) complex against HT-29 cells. The result showed significant cytotoxicity at concentration of 3 μg/mL. The data represent the means ± SD of three independent experiments. *P < 0.05 compared with the no-treatment group.

The release of the lactate dehydrogenase (LDH) enzyme as a biomarker suggests the loss of membrane integrity, apoptosis, or necrosis. The cytotoxic effects of the CdCl_2_(C_14_H_21_N_3_O_2_) complex on HT-29 cells, which were treated with the complex for 48 h, were assessed by LDH assay, and the results demonstrated a significant increase in the level of LDH release, i.e., cytotoxicity, at concentration of 3 μg/mL compared with the control cells (Figure 1).

### CdCl_2_(C_14_H_21_N_3_O_2_) complex induces G_1_ cell cycle arrest

**Figure 2:**
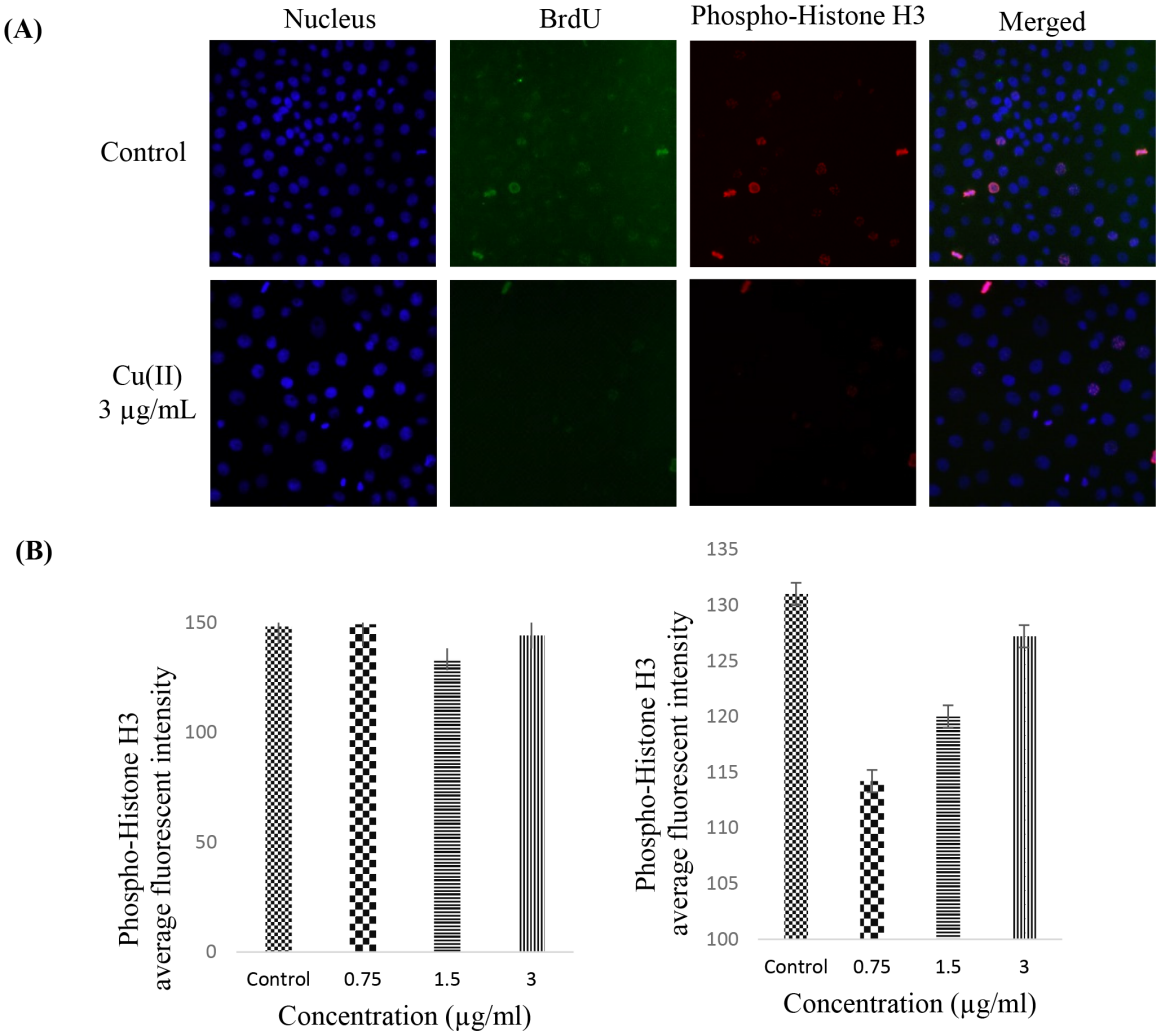
Activity of CdCl_2_(C_14_H_21_N_3_O_2_) complex on cell cycle arrest in the S/M phase. (A) After incubation with DMSO (negative control) or Schiff based compound (3 μg/mL) for 24 h, HT-29 cells were collected, stained with BrdU (representing the S phase) and Phospho-Histone H3 (representing the M phase), and subjected to cell cycle analysis using a Cellomics ArrayScan HCS reader. (B) Representative bar charts showing that treatment with CdCl_2_(C_14_H_21_N_3_O_2_) complex caused no significant changes in the BrdU and Phospho-Histone H3 fluorescence intensities, suggesting that the cells do not arrest at the S/M phases. The data represented the means ± SD of the fluorescence intensity readings from three independent experiments. *P < 0.05 compared with the no-treatment group.

**Figure 3:**
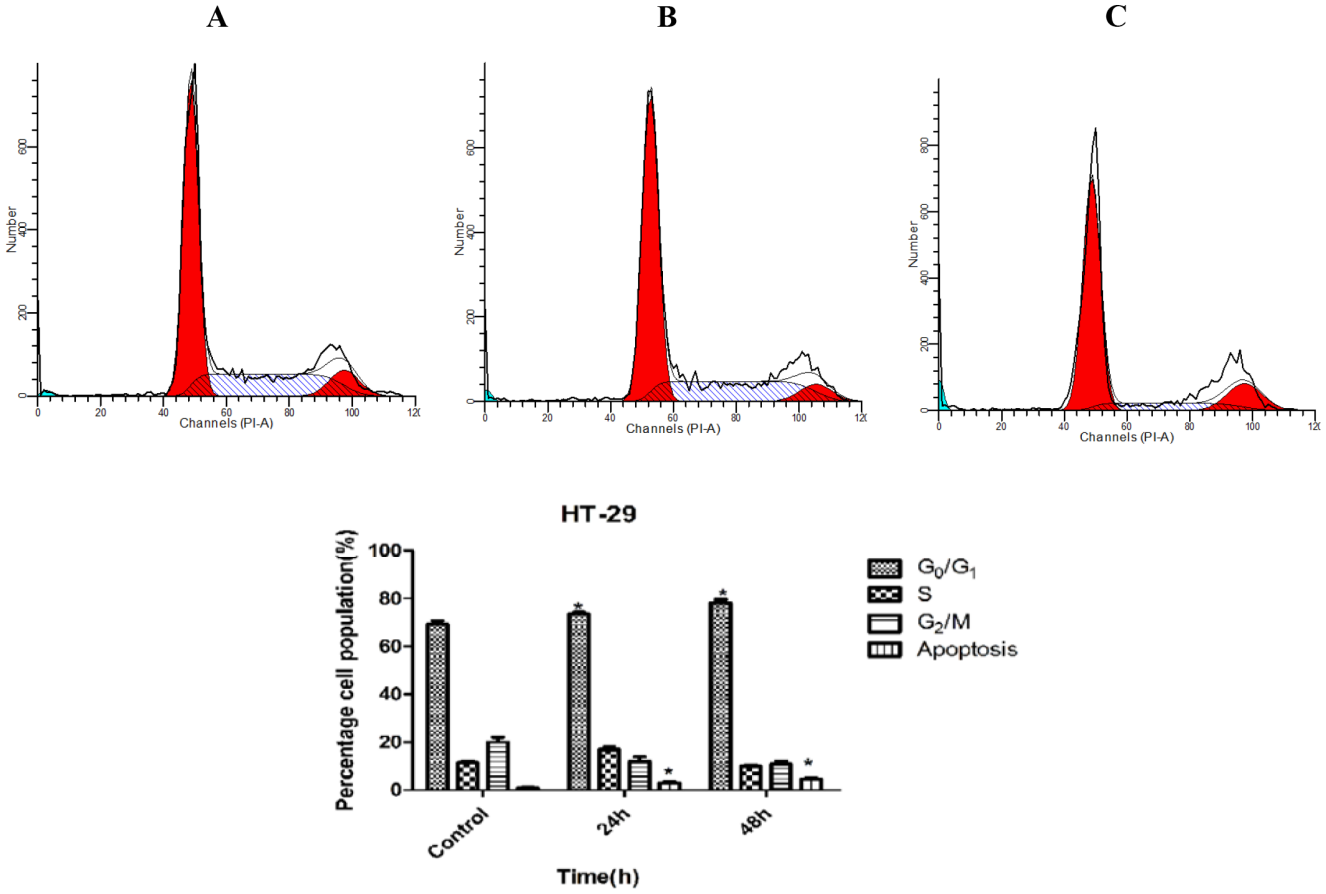
Effect of CdCl_2_(C_14_H_21_N_3_O_2_) complex on cell cycle progression in HT-29 cells. This effect was assessed by flow cytometry. After incubation with the Schiff based compound for 24 and 48 h, significant cell cycle arrest at the G_1_ phase was observed. All of the data are expressed as the means ± standard error of triplicate measurements. *P < 0.05 compared with the no-treatment group.

A dysfunction of the cell cycle regulation that leads to the overproliferation of normal cells is a key factor in the development of cancer. Thus, the suppression of the cell cycle machinery in cancer cells can strongly limit cancer progression. Therefore, we investigated the effect of the 3 μg/ml CdCl_2_(C_14_H_21_N_3_O_2_) complex on the cell cycle distribution. As illustrated in Figure 2, the BrdU and Phospho-Histone H3 staining of HT-29 cells treated with the complex demonstrated no cell cycle arrest at the S/M phases. In addition, the flow cytometry data show the complementary results of the cellular arrest in the G_1_ phase (Figure 3).

### Quantification of apoptosis using phase-contrast microscopy and AO/PI double-staining

**Figure 4:**
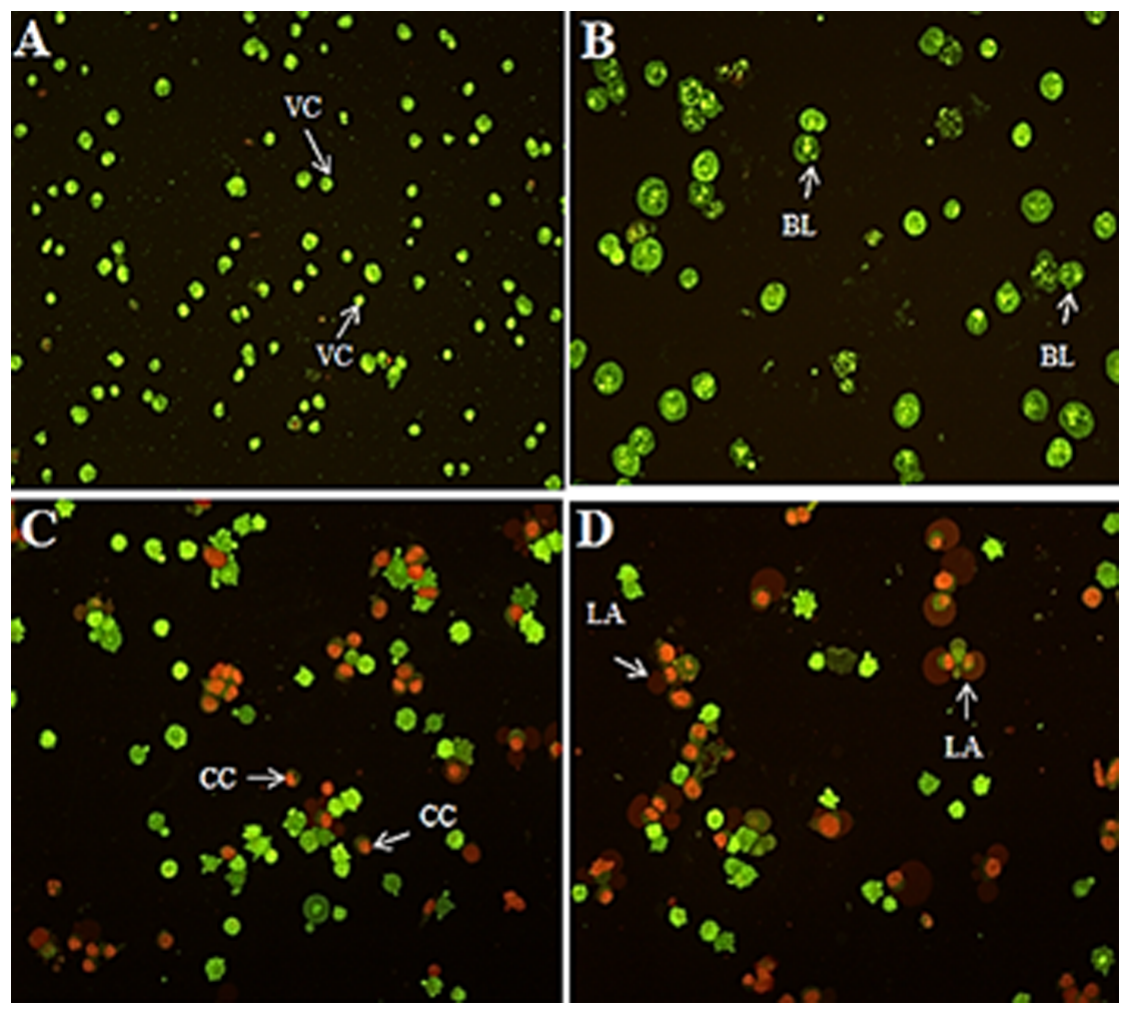
Fluorescent micrographs of acridine orange and propidium iodide double-stained HT-29 cells. (A) Untreated HT-29 cells were healthy after 72 h. In addition, early apoptotic features, including blebbing and chromatin condensation, were observed after (B) 24 and (C) 48 h. (D) Late apoptosis events were observed after 72 h of treatment with 3.0 μg/ml CdCl_2_(C_14_H_21_N_3_O_2_) complex (magnification: 200×).VC: Viable cells; BL: Blebbing of cell membrane; CC: Chromatin condensation; LA: Late apoptosis.

The apoptotic properties of HT-29 cells treated with 3 μg/ml CdCl_2_(C_14_H_21_N_3_O_2_) complex were detected under a fluorescent microscope after 24, 48, and 72 h of treatment. The control cells presented intact green fluorescence, showing the normal nuclear structures. Bright green fluorescence with intervening acridine orange (AO) within the fragmented DNA was found as an early apoptotic feature. After 24 and 48 h, nuclear chromatin condensation and membrane blebbing were detected as moderate apoptotic characterisations. Moreover, after 72 h of treatment with the complex, the presence of a reddish-orange colour due to the binding of PI to denatured DNA wan observed, presenting the late stage of apoptosis (Figure 4).

### Reactive oxygen species (ROS) generation

**Figure 5:**
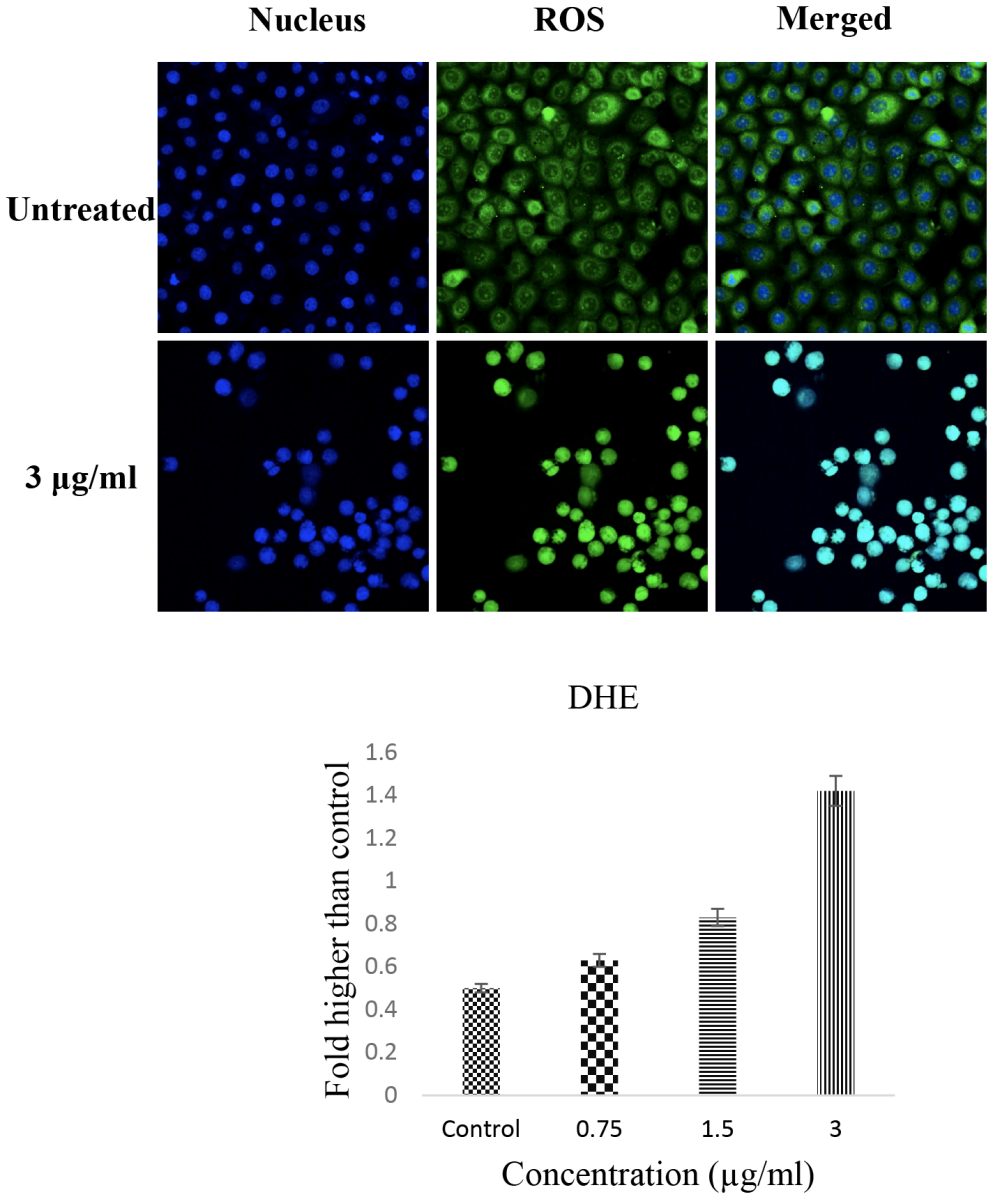
ROS generation in the presence of CdCl_2_(C_14_H_21_N_3_O_2_) complex. At concentration of 3 μg/mL, the Schiff based compound caused significant ROS formation in HT-29 cells. All of the data are expressed as the means ± standard error of triplicate measurements. *P < .05 compared with the no-treatment group.

The generation of reactive oxygen species (ROS) plays a critical role in the activation of mitochondrial-initiated events leading to apoptosis. The ROS scavenging antioxidant system can be disrupted by an increased level of intracellular ROS. In our experiment, the results of the ROS assay revealed the oxidation of dihydroethidium (DHE) to ethidium in the presence of the Schiff based compound after 24 h of treatment. As shown in Figure 5, the CdCl_2_(C_14_H_21_N_3_O_2_) complex at concentrations of 3 μg/mL induced a significant increase in the level of generated ROS.

### CdCl_2_(C_14_H_21_N_3_O_2_) complex induced MMP perturbation and cytochrome c release

**Figure 6:**
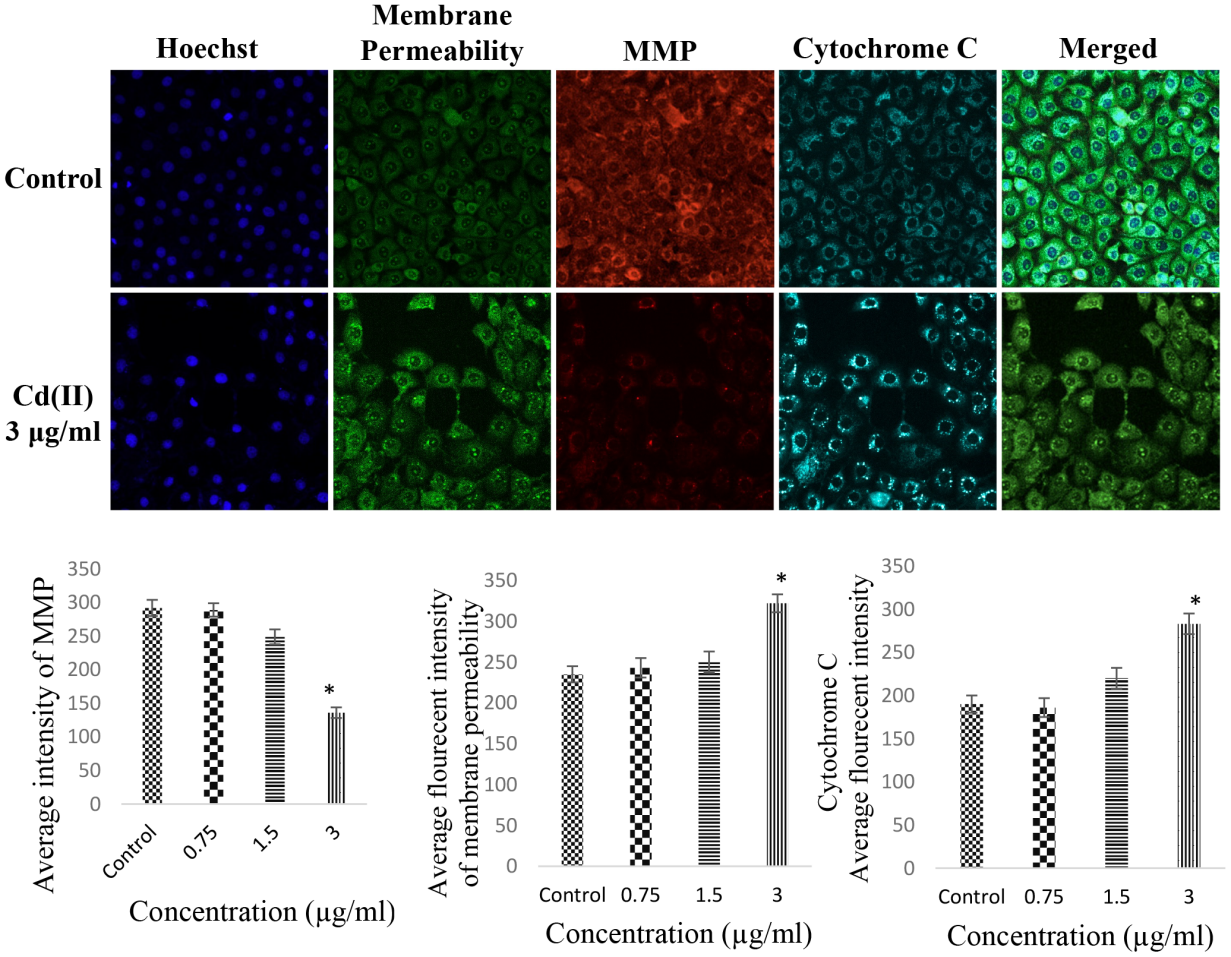
Effects of CdCl_2_(C_14_H_21_N_3_O_2_) complex on nuclear morphology, membrane permeability, mitochondrial membrane potential (MMP) and Cytochrome c release. (A) Representative images of HT-29 cells stained with Hoechst 33342, cytochrome *c*, membrane permeability, and MMP dyes after treatment with 3 μg/mL CdCl_2_(C_14_H_21_N_3_O_2_) complex (magnification: 20×). (B) Representative bar charts indicating the dose-dependent reduction in MMP, the increased cell permeability, and the cytochrome *c* release in treated HT-29 cells. All of the data are expressed as the means ± standard error of triplicate measurements. *P < 0.05 compared with the no-treatment group.

Hoechst 33342 staining revealed the nuclear condensation of some HT-29 cells after treatment with CdCl_2_(C_14_H_21_N_3_O_2_) complex, as represented by apoptotic chromatin changes. The quantitative analysis of multiple cytotoxicity assays also revealed significant elevations in the levels of cell permeability, MMP, and cytochrome *c* release (Figure 6). The survival and death of cells is closely regulated by the mitochondria, as the main producer of ROS and adenosine triphosphate (ATP). Thus, any changes in the regulated level of MMP can lead to the activation of apoptosis and the release of pro-apoptotic factors, such as cytochrome *c*.

### Caspase activation

**Figure 7:**
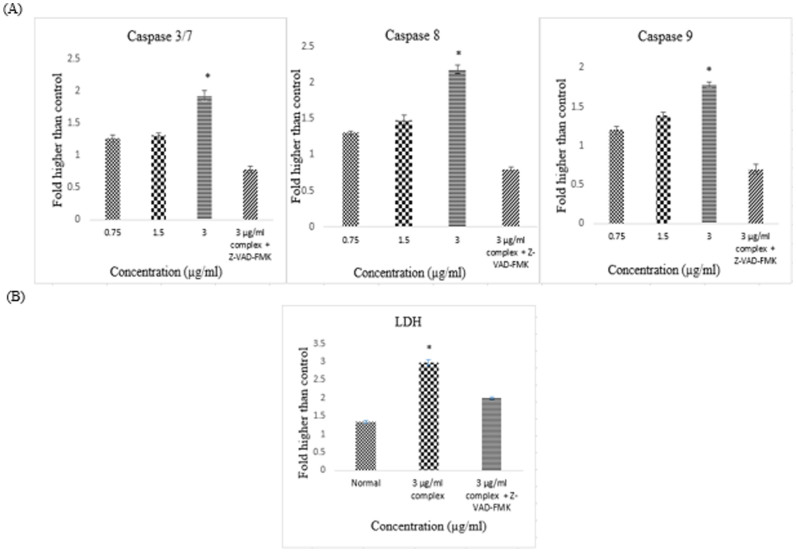
Effect of various concentrations of CdCl_2_(C_14_H_21_N_3_O_2_) complex on caspase 3/7, 8, and 9 activation in HT-29 cells after 24 h of treatment. The results revealed significant activation of caspases-3/7, -8, and -9. All of the data are expressed as the means ± standard error of triplicate measurements. *P < 0.05 compared with the no-treatment group.

To check whether caspases were involve in the induction of apoptosis, caspase-3/7, -8, and -9 activities were quantified in HT-29 cells treated with 0.75, 1.5 and 3 μg/ml of CdCl_2_(C_14_H_21_N_3_O_2_) complex at different concentrations. As illustrated in Figure 7, the activation of caspase-3/7 and -9 was obtained after treatment with the CdCl_2_(C_14_H_21_N_3_O_2_) complex at concentration of 3 μg/ml, respectively. Furthermore, caspase-8 was activated after treatment with the Schiff based complex at similar concentration. In contrast, addition of pan caspase inhibitor, Z-VAD-FMK attenuated caspase 3/7, 8, 9 activities in the presence of 3 μg/ml CdCl_2_(C_14_H_21_N_3_O_2_) complex.

### NF-κB Translocation

**Figure 8:**
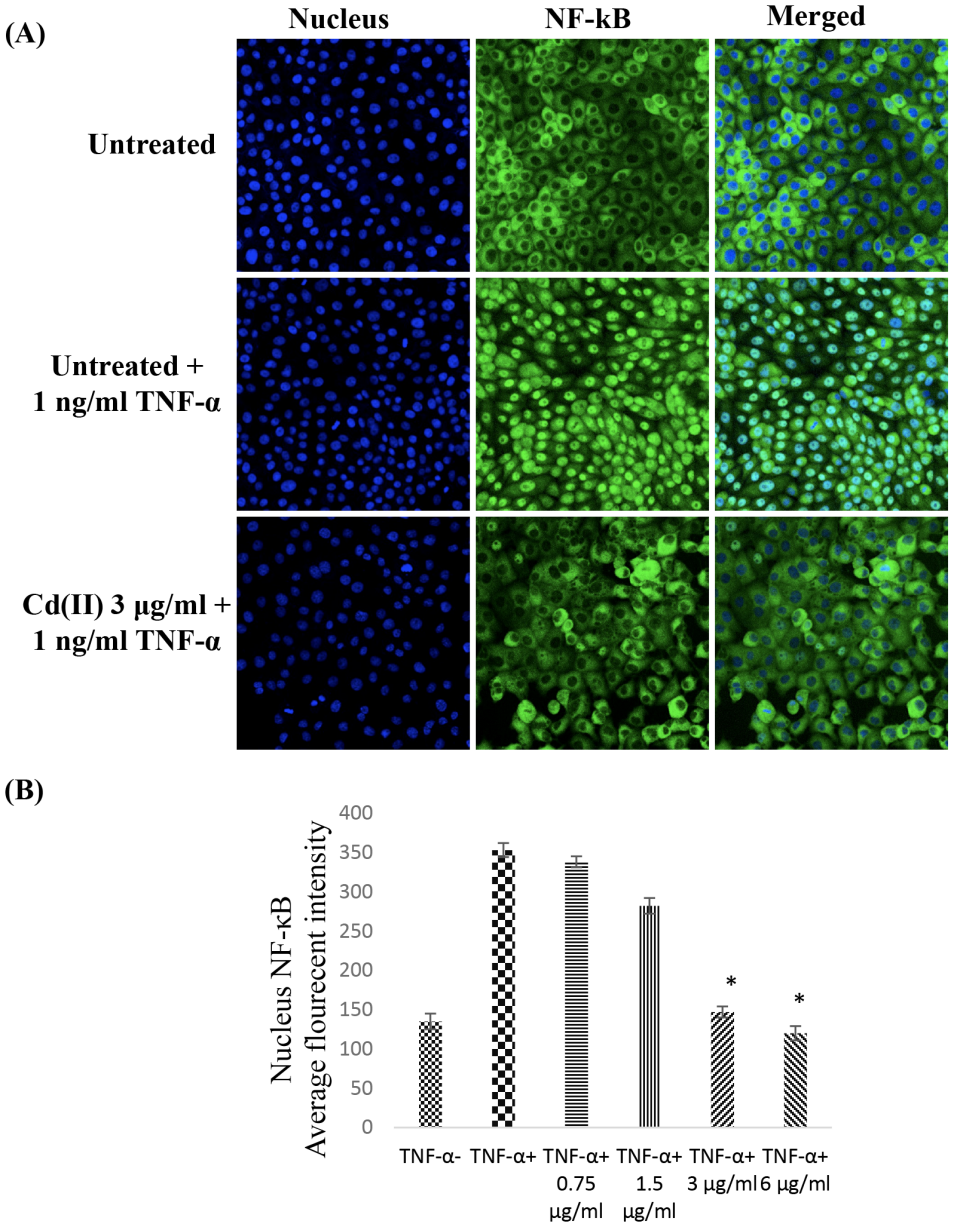
NF-κB Translocation. (A) Images and (B) representative bar chart of HT-29 cells after treatment with various concentrations of CdCl_2_(C_14_H_21_N_3_O_2_) complex for 3 h and subsequent exposure to TNF-α (1 ng/mL) as an NF-κB activator for 30 min. The results did not reveal any significant translocation of NF-κB from the cytoplasm to the nucleus. All of the data are expressed as the means ± standard error of triplicate measurements. *P < 0.05 compared with the no-treatment group.

The transcription factor of nuclear factor kappa B (NF-κB) plays a critical role in the control of the transcription of DNA and the regulation of harmful cellular stimuli. Inflammatory cytokines of tumour necrosis factor-α (TNF-α), which is the known activator of this protein complex, can facilitate the translocation of NF-κB from the cytosol to the nucleus and the induction of its DNA-binding activity. The CdCl_2_(C_14_H_21_N_3_O_2_) complex at a concentration of 3 μg/mL demonstrated a significant suppressive effect against the translocation of TNF-α-stimulated NF-κB in HT-29 cells (Figure 8). The cytoplasm of the control cells illustrated high NF-κB fluorescent intensity, representing the non-stimulated condition. In addition, the HT-29 cells stimulated with TNF-α exhibited significant fluorescent intensity in the nucleus, and this fluorescence was apparently reduced after treatment with the CdCl_2_(C_14_H_21_N_3_O_2_) complex.

### Western blot analysis

**Figure 9:**
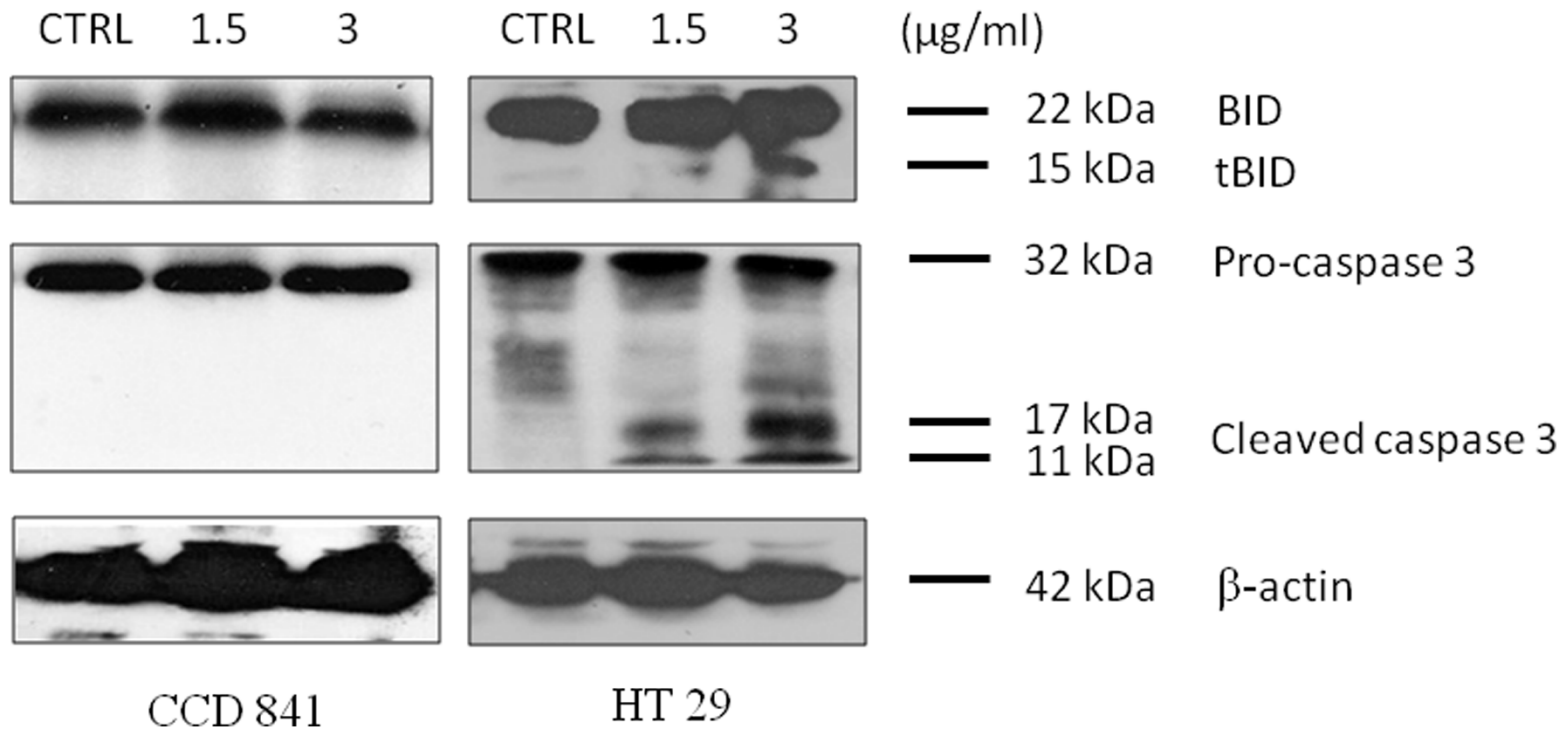
Western blot analysis of CdCl_2_(C_14_H_21_N_3_O_2_)-treated HT-29 cells. Western blot analysis revealed the expression levels of cleaved caspase-3 and truncated Bid in CdCl_2_(C_14_H_21_N_3_O_2_)-treated HT29 and CCD841 cells. β-actin served as a loading control.

Cleavage of cytosolic pro-apoptotic factor Bid by activated caspase-8 leads to its truncation (tBid) after induction of cytochrome c release. To examine this, HT29 or CCD841 cells were treated with 1.5 or 3 μg/mL of CdCl_2_(C_14_H_21_N_3_O_2_) for 48 h and subjected to western blotting analysis. As illustrated in Figure 9, the expression of truncated Bid (tBid; 15 kDa) only appear in complex treated HT29 cells, whereas only total Bid (22 kDa) was detected in normal CCD841 colon cells treated with 1.5 or 3 μg/mL of CdCl_2_(C_14_H_21_N_3_O_2_). In addition, cleaved caspase 3 (17 and 11 kDa subunits) was detected in complex treated HT29 cells, but not in normal CCD841 colon cells treated with the similar dosages. Therefore, the results showed that CdCl_2_(C_14_H_21_N_3_O_2_) has a role on induction of apoptosis by caspase-8 which leads to the formation of tBid, followed by the activation of downstream caspase 3. Moreover, these dosages did not induce activation of caspases (8 or 3/7) in normal CCD841 normal colon cells.

### Annexin-V-FITC assay

**Figure 10:**
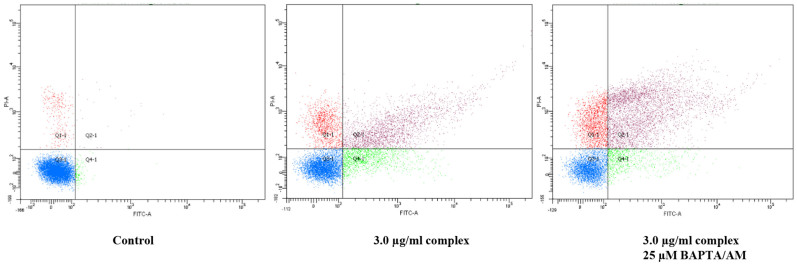
Apoptosis evaluation of HT-29 cells treated with Calcium cheator BAPTA/AM piror to CDCL2(C14H21N3O2) compound. (A) Represents the untreated cells as the control, (B) 24 h treatment of HT-29 cell with compound, (C) 24 h treatment of HT-29 cell with BAPTA/AM and CDCL2(C14H21N3O2) compound.

HT29 cells were pretreated with cell membrane permeable calcium chelator (BAPTA/AM, Sigma, 25 μM) for 1 hour followed by addition of complex (3.0 μg/ml) for 24 hours. Cells were stained with Annexin V FITC and PI then subjected to flow cytometry analysis. As shown in figure 10, our data indicated that Ca2+ depletion did not inhibit complex-induced apoptosis.

### The expression of Bcl-2 and Bax

**Figure 11:**
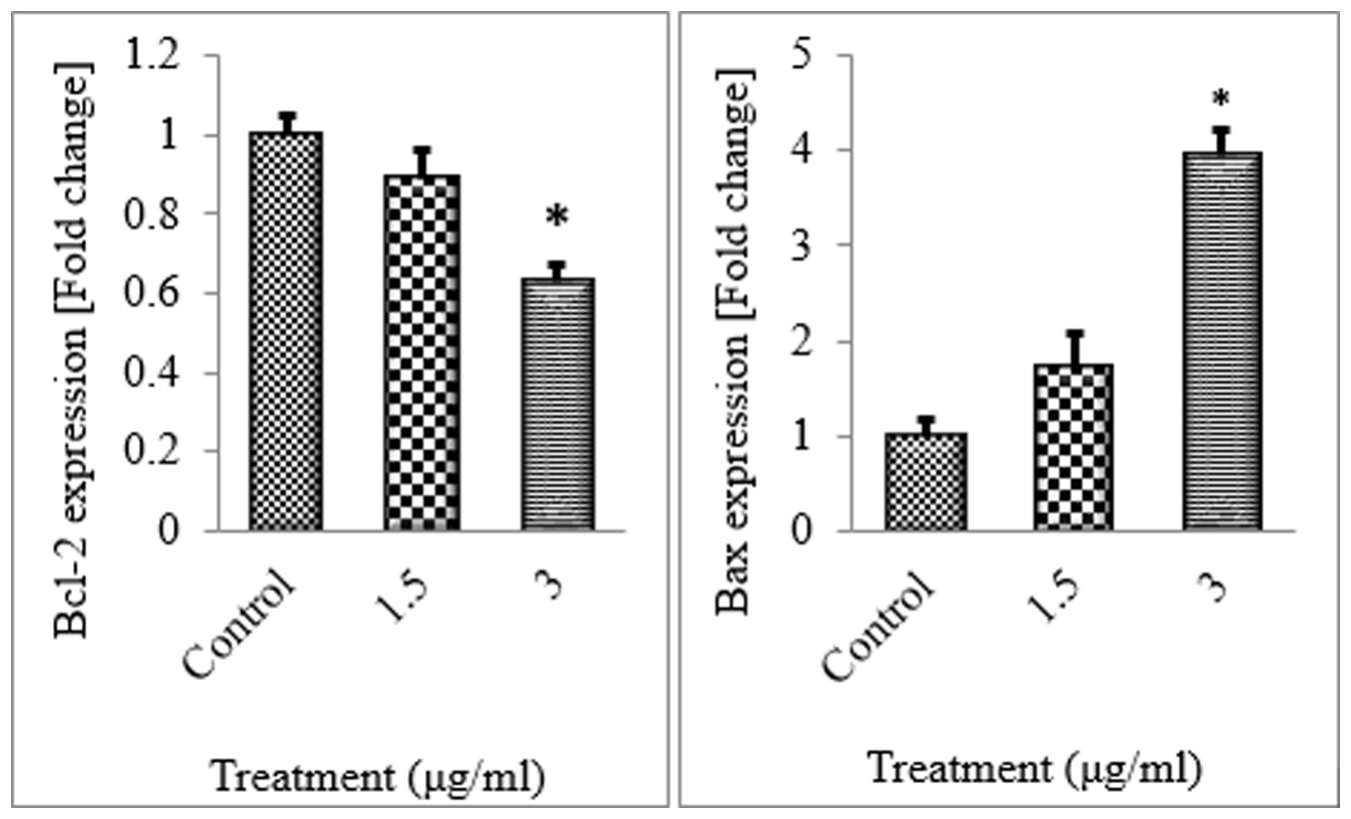
HT-29 cells were treated with DMSO or different concentrations of CdCl_2_(C_14_H_21_N_3_O_2_) complex for 12 hours. RNAs were isolated and converted to cDNA. Quantitative real-time PCR was performed to determine expression level of Bcl-2, Bcl-xl and Bax genes. GAPDH was used as a housekeeping gene.

Bcl-2 family of proteins regulate Mitochondrial Outer Membrane Permeabilization (MOMP). They include anti-apoptotic molecules such as Bcl-2, which could preserve cell survival and pro-apoptotic molecules like Bax that inhibit cell survival. To examine whether the expression of these molecules were affected at the transcriptional level, we performed quantitative real-time PCR using untreated or CdCl_2_(C_14_H_21_N_3_O_2_)-treated colon cancer cells. The results indicated a marked increase in Bax expression, but decrease in the expression level of Bcl-2 in the treated HT-29 cells (Figure 11). Supplementary table S1 showes corresponding assays, cell number of each assay, complex concentrations and the corresponding tables and figures.

## Discussion

Cancer incidence is a result of non-balanced hemostasis between cell proliferation and cell death in multicellular organisms. The cell death program plays a controlling role from the outer membrane of undesirable cells to each important organelle inside the cell, such as the mitochondria and chromatin^18^,^19^. With the increasing range of cancers and valuable studies on transitional metal chemistry and drug discovery, the biological activity of various synthetic Schiff based compounds has been studied to determine their role in the induction of cancer cell apoptosis and to identify the key events and molecules that are regulated in the pathway^20^,^21^. The aim of this study was to determine whether the CdCl_2_(C_14_H_21_N_3_O_2_) complex has an effect on the activation of the apoptotic pathway in a colon cancer cell line. The cell line model used in this study was HT-29, which was recently used as a model in different studies^22^,^23^. The MTT cytotoxic assay showed a dose- and time-depended growth inhibition of HT-29 cells with an IC_50_ value of 2.57 μg/mL after 72 h of treatment with the CdCl_2_(C_14_H_21_N_3_O_2_) complex. The safety characterisation of the complex was confirmed, and no cytotoxic effect was observed after treatment of a normal colon cell line (CCD 841) with different concentrations of the complex. The LDH measurement assay confirmed the cytotoxic effect of the complex through the activation of apoptosis or necrosis^24^. In this experiment, the increase in the level of LDH after treatment with the complex at concentrations of 3 μg/mL indicates that the cytotoxicity is a result of membrane integrity damage. The analysis of the early and late apoptosis characteristics of HT-29 cells treated with the CdCl_2_(C_14_H_21_N_3_O_2_) complex through AO/PI double-staining revealed some qualitative morphological proof of apoptosis, such as cytoplasmic shrinkage, membrane blabbing, and DNA fragmentation^25^. An increase in the treatment duration from 24 to 72 h revealed a change in the apoptosis features from early to late apoptosis, which revealed the possibility of necrosis in cells exposed to the complex for a longer period of time. Based on the AO/PI dual staining assay, healthy viable cells were observed in the untreated cell samples. To further confirm the accruing of apoptosis, the cell cycle distribution was analysed through the BrdU and Phospho-Histone H3 staining of HT-29 cells treated with the CdCl_2_(C_14_H_21_N_3_O_2_) complex^26^,^27^,^28^. The photographs illustrate that there was no attachment of BrdU to DNA and no H3 staining of the cells in the mitotic stage. Thus, no significant difference in the number of cells in the S/M phases was found, which indicates that the cells were arrested at the G_1_ or G_2_ phases, which is a marker of cell death caused by apoptosis; this finding was confirmed through a flow cytometry assay^29^,^30^. The increasing levels of ROS, which is a factor that triggers apoptosis, in HT-29 cells after treatment with the CdCl_2_(C_14_H_21_N_3_O_2_) complex prompted us to examine the mitochondrial membrane potential (MMP) in these cells using fluorescent probes. The mitochondria play an important role in the regulation of cell death and survival^31^. The reaction of ROS with phospholipids of the mitochondrial membrane results in the opening of transition pores in the early stage of apoptosis^32^,^33^ and thus decreases the membrane permeability. Damage to the potential efficiency of the mitochondrial membrane occurs especially in reference to its vital role in apoptosis in the presence of metals as an inducer^34^. The decrease in the cytosolic cytochrome *c* level in the mitochondria is a sign of apoptosis initiation^35^. The release of rhodamine 123 from the mitochondrial matrix to the cytoplasm as a result of membrane depolarisation and the increase in the level of cytochrome *c* after exposure of the cells to CdCl_2_(C_14_H_21_N_3_O_2_) complex implies that the observed apoptosis is induced via the intrinsic mitochondrial pathway^36^,^37^. The experimental data demonstrate that the increase in the level of intracellular ROS after the treatment of HT-29 cells with CdCl_2_(C_14_H_21_N_3_O_2_) complex caused mitochondrial dysfunction and increased the level of cytochrome *c* in the cytosol, which indicates the activation of caspase molecules via binding to apoptotic activating factor-1. There are two types of intrinsic and extrinsic apoptotic pathways. Caspase-3/7, as downstream executioner caspases, would be activated as a result of the activation of caspase-8^38^,^39^. A significant elevation in the caspase-8 pathway was obtained after the treatment of HT-29 cells with the CdCl_2_(C_14_H_21_N_3_O_2_) complex, and the caspase-9 activation demonstrated that more than one pathway is involved in the apoptosis exerted by the CdCl_2_(C_14_H_21_N_3_O_2_) complex as an anticancer agent. Following treatment of cytotoxic drugs the extrinsic pathway of apoptosis which is usually known as death receptor-dependent apoptosis such as CD95/Fas, IL-R, TNF-R, lead to activation of caspase 8 which play role in trigger of others caspases whether through intrinsic pathway by cleavage of Bid or other caspase which are non-depended on intrinsic pathway^40^,^41^. However according to other study some drugs induced activation of caspase 8 while there was no interaction of death domain adaptor protein and ligand and there is intercede of intrinsic apoptosis signaling pathway on activation of caspase 8^42^.

One of the caspase-8 substrate named Bid that following translocation of tBid to mitochondria, it leads to release of cytochrome c and activation of Bax^43^. According to the current experiment caspase-8/Bid pathway induced apoptosis following treatment of HT29 cells with the complex. The pervious studies showed applying calcium chelator BAPTA was inhibited caspase 9 activation while there was no interference was observed on stimulation of the extrinsic pathway of apoptosis through caspase 8^44^,^45^. Based on the result of current study on flow cytometry analysis of Annexin-V we found an elevation in the number of cells which went through apoptosis following treatment of compound although the cytosolic free calcium was not exist as a result of calcium chelator BAPTA which was added perior to treated HT29 cell with compound. So its bring the idea that the CDCL2(C14H21N3O2) compound trigger caspase 8 which play important role on inducing apoptosis aside from intracellular calcium concentrations^46^. Based on perevious studies Bcl2 protein family play important role to mediate cytochrome c release in the context of apoptotic stimuli. These proteins play important parts in the improvement of novel cancer drugs. Bcl-2 by blocking various apoptosis signals plays an essential role to controll the process of cell death. The Bax protein has a role in the release of a factor that stimulates apoptosis into the cytoplasm. Therefore, the balance of the expressions of these proteins is important in the process of cell death^47^,^48^.The expression level of BAX was upregulated following treatment of colon cells with the complex. Our results showed that CdCl_2_(C_14_H_21_N_3_O_2_) cause a significant decrese in the expression level of the Bcl2 protein. The oncolysis induced by CdCl_2_(C_14_H_21_N_3_O_2_) through apoptosis, so pointed to the association of Bax and Bcl2 at translational level. This study provided evidence that the CdCl_2_(C_14_H_21_N_3_O_2_) complex may play an anticancer role against HT-29 cells by decreasing the activation of the NF-κB signalling pathways. Several studies have shown the pivotal regulatory role of NF-κB signalling in different cancer cells. They have described the role of this signalling pathway in the resistance of tumours cells against anticancer drugs^49^. Cell proliferation is suppressed in response to the activation of an inhibitor of NF-κB that prevents its binding to DNA. Therefore, agents that can regulate the NF-κB signalling pathway may be notable chemo-therapeutic targets in cancer therapy^36^,^49^,^50^.

## Conclusions

The supporting evidence of LDH release, ROS production, MMP suppression, elevation of the level of cytochrome *c*, and activation of caspase-9 and -8 after suppression of the NF-κB signalling pathway demonstrate the promising anticancer activity of the CdCl_2_(C_14_H_21_N_3_O_2_) complex against the HT-29 colon cancer cell line via both intrinsic and extrinsic mitochondrial pathways.

## Methods

### Reagents

All of the chemicals were achieved from Sigma-Aldrich (St. Louis, MO, USA). Stock solutions of the tested compound were prepared in dimethyl sulfoxide (DMSO) and stored at −20°C in the dark.

### Test material

**Figure 12:**
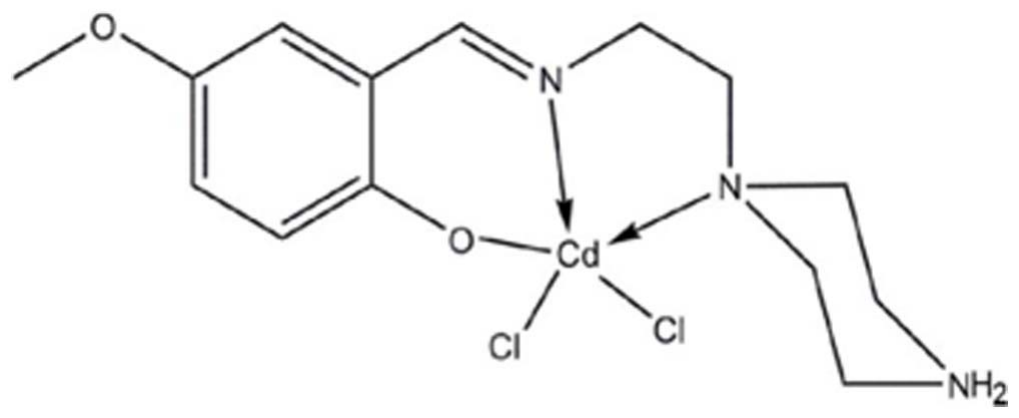
Chemical structure of CdCl_2_(C_14_H_21_N_3_O_2_).

As described previously, Dichlorido(4-methoxy-2-{[2-(piperazin-4-ium-1-yl)ethyl]iminomethyl} phenolate) cadmium complex (CdCl_2_(C_14_H_21_N_3_O_2_)) (Figure 12) was kindly supplied by Prof. Dr. Hapipah Mohd Ali, Department of Chemistry, Faculty of Science, University of Malaya, Kuala Lumpur, Malaysia^51^.

### Cell culture and cell viability assay

Normal human colon epithelial cells (CCD 841 cells) and human colon cancer cells (HT-29 cells) were obtained from ATCC (Manassas, VA, USA) and maintained in RPMI 1640 supplemented with heat-inactivated FBS (10%), streptomycin (100 μg/mL), and penicillin G (100 U/mL). The cells were cultured at 37°C in a humidified atmosphere of 95% air/5% CO_2_. The cell viability was analysed by MTT [3-(4,5-dimethylthiazol-2-yl)-2,5,-diphenyltetrazolium bromide] assay. After 24, 48, and 72 hours, the cells treated with the CdCl_2_(C_14_H_21_N_3_O_2_) complex were stained with MTT solution (10 mL; 5 mg/mL in phosphate-buffered saline) for 3 h to dissolve the dark formazan crystals. The absorbance was then measured at 570 nm using a microplate reader (Hidex, Turku, Finland). The IC_50_ value was evaluated as the concentration of the complex required to reduce the absorbance of the treated cells to 50% of that of the DMSO-treated control cells. All of the samples were prepared in triplicate.

### LDH release assay

To assess the cytotoxicity potential of the CdCl_2_(C_14_H_21_N_3_O_2_) complex, the lactate dehydrogenase (LDH) release assay was performed. HT-29 cells were treated with the complex (0, 0.75, 1.5 and 3 μg/ml) for 48 h. and the supernatant of the cells was transferred to a new 96-well plate. After adding the LDH reaction solution (100 μL), the plate was incubated for 30 min. The absorbance was then read at 490 nm using a Tecan Infinite®200 Pro (Tecan, Männedorf, Switzerland) microplate reader. The amount of formazan salt and the intensity of the red colour in the samples represented the LDH activity.

### Cell cycle analysis

The cell cycle distribution of the HT-29 cells was analysed using a fluorescence microscope. Briefly, HT-29 cells were treated with 3 μg/mL CdCl_2_(C_14_H_21_N_3_O_2_) complex or DMSO as negative control for 24 h. Then, BrdU and Phospho-Histone H3 dyes were added to the treated cells for 30 min. After fixation, the cells were observed and analysed using a Cellomics ArrayScan HCS reader (Thermo Scientific). The target activation bioapplication module was applied to measure the fluorescence intensities of the dyes.

A flow cytometry assay was conducted to confirm the fluorescence microscopy results. After incubation with the complex (3 μg/mL) for 24, 48, and 72 h, the HT-29 cells were centrifuged at 1800 rpm for 5 min. To restore their integrity, the cell population was fixed for flow cytometry analysis. Briefly, the cell pellets were mixed with 700 μL of cold 90% ethanol and then maintained at 4°C overnight. After washing and suspending the cells in PBS, 25 μL of RNase A and 50 μL of PI were added to the fixed cells, and the mixture was incubated for 1 h at 37°C. At the end of the incubation period, the DNA content of the cells was analysed using a flow cytometer (BD FACSCanto™ II).

### Acridine orange/ propidium iodide double staining

To detect the early and late apoptotic properties of the treated HT-29 cells, a propidium iodide (PI) and acridine orange (AO) double staining assay was performed using a fluorescent microscope (Leica attached with Q-Fluoro software) according to the standard procedure. Briefly, the cells were incubated with 3 μg/mL CdCl_2_(C_14_H_21_N_3_O_2_) complex for 24, 48, and 72 h. The harvested cells were then stained with the AO/PI fluorescent dyes and observed under a UV-fluorescent microscope (Olympus BX51) within 30 min.

### Measurement of reactive oxygen species (ROS) generation

HT-29 cells (1 × 10^4^ cells/mL) were treated with CdCl_2_(C_14_H_21_N_3_O_2_) complex (0, 0.75, 1.5 and 3 μg/ml) or DMSO (negative control) in a 96-well plate for 24 h. The treated cells were stained with dihydroethidium (DHE) dye for 30 min. In the presence of superoxides, DHE dye is oxidised to ethidium. The fluorescence intensity was measured using a fluorescent plate reader using an excitation wavelength of 520 nm and an emission wavelength of 620 nm.

### Multiple cytotoxicity assay

The crucial factors involved in programmed cell death, namely cell loss, changes in cell permeability, cytochrome *c* release, changes in the mitochondrial membrane potential (MMP), changes in nuclear size, and morphological changes, were evaluated using a Cellomics Multiparameter Cytotoxicity 3 Kit. The plates with the stained cells were analysed using the ArrayScan HCS system (Cellomics, PA, USA).

### Measurement of caspase activities

To measure the activities of the caspases, HT-29 cells were treated with CdCl_2_(C_14_H_21_N_3_O_2_) complex (0, 0.75, 1.5 and 3 μg/ml) and pan caspase inhibitor, Z-VAD-FMK for 48 hours.for 24 h and then analysed using the commercial Caspase-Glo® 3/7, 8, and 9 assay kit (Promega, Madison, WI, USA). In the case of caspase activation in apoptotic cells, the substrate of the luciferase enzyme would be released after the cleavage of the aminoluciferin-labelled synthetic tetrapeptide. The caspase activities were analysed using a Tecan Infinite®200 Pro (Tecan, Männedorf, Switzerland) microplate reader.

### Measurement of NF-κB activity

HT-29 cells treated with CdCl_2_(C_14_H_21_N_3_O_2_) complex (3 μg/mL) and then stimulated with TNF-α were stained according to the instructions of the manufacturer of the Cellomics nucleus factor-κB (NF-κB) activation kit (Thermo Scientific). The cytoplasm to nucleus translocation bioapplication software was used to measure the cytoplasmic and nuclear NF-κB intensity ratio (average intensity of 200 cells/well).

### Western blot analysis

To prepare the samples, cells were washed twice with cold PBS and suspended in RIPA lysis buffer (Santa Cruz Biotechnology, Santa Cruz, CA) supplied with the protease inhibitors. 30 mg of the extracted protein were loaded onto 10% polyacrylamide gel and then transferred to a PVDF membrane (Millipore, Billerica, MA). The membranes were blocked with 5% bovine serum albumin (BSA) for an hour, then immunoblotted with anti-Bid (1:1000), anti-procaspase-3 (1:300) or anti-β-actin (1:10,000) primary antibodies (Cell Signaling Technology, Beverly, MA) overnight. After three times washing with PBS, the membranes were incubated with horseradish peroxidase (HRP)-conjugated secondary antibodies (Santa Cruz Biotechnology). The signal was detected using ECL Plus Chemiluminescence Reagent according to the manufacturer's instruction (Amersham, Chalfont, UK).

### Annexin-V-FITC Assay

The cells were seeded into a chamber slide plate and were pretreated with cell membrane permeable calcium chelator (BAPTA/AM, Sigma, 25 μM) for 1 hour prior to addition of complex. After 24 h of CDCL2(C14H21N3O2) (3.0 μg/ml) treatment, The adherent and suspended cells were harvested and washed twice with PBS. Then, the HT29 cells were then re-suspended in Annexin-V binding buffer (BD Biosciences, San Jose, CA, USA) and stained with Annexin-V-FITC (BD) and PI (Sigma) according to the vendor's instructions. The fluorescent intensity of HT29 cells was then examined using flow cytometry (BD FACSCanto™ II) and quadrant statistics for necrotic and apoptotic cell populations. Detection of early and late apoptosis was done by Annexin-V, while PI was responsible for the detection of late apoptosis and necrosis.

### Quantitative PCR analysis

HT-29 cells were treated with different concentrations of CdCl_2_(C_14_H_21_N_3_O_2_) complex (0, 1.5 and 3 μg/ml) for 12 hours. Zymo Research Quick-RNA™ MiniPrep kit was used to isolate the total RNAs and complimentary DNAs were synthesized using Applied Biosystems High Capacity RNA-to-cDNA™ Kit. Quantitative PCR was carried out on Applied Biosystems StepOnePlus™ system using Applied Biosystems TaqMan® Fast Advanced Master Mix and TaqMan® Gene Expression Assays. The obtained data were then normalized to GAPDH. The IDs for TaqMan® Gene Expression Assays used in this experiment are listed in Table 2.

### Statistical Analysis

Each assay was performed three times independently. Analysis of variance (ANOVA) was conducted using the Prism statistical package (GraphPad Software, USA). The results are presented as the means ± standard deviation (SD) of the number of experiments. P < 0.05 was considered statistically significant.

## Supplementary Material

Supplementary Information Supplementary Table S1 corresponding assays, cell number of each assay, complex concentrations and the corresponding tables and figures.

## Figures and Tables

**Table 1:** Cytotoxic effect of CdCl_2_(C_14_H_21_N_3_O_2_) complex against HT-29 and CCD 841 cell lines after 24, 48, and 72 h

Cell line	Classification	IC_50_ (μg/mL)		
		24 h	48 h	72 h
HT-29	Colon cancer cells	3.49 ± 0.52	2.83 ± 0.64	2.57 ± 0.39
CCD 841	Normal colon cells	50 <	50 <	50 <

The IC_50_ values were determined through non-linear regression analysis.

**Table 2:** IDs for TaqMan® Gene Expression Assays

Target gene	Assay ID
GAPDH	Hs02758991_g1
Bcl-2	Hs00608023_m1
Bax	Hs00180269_m1
