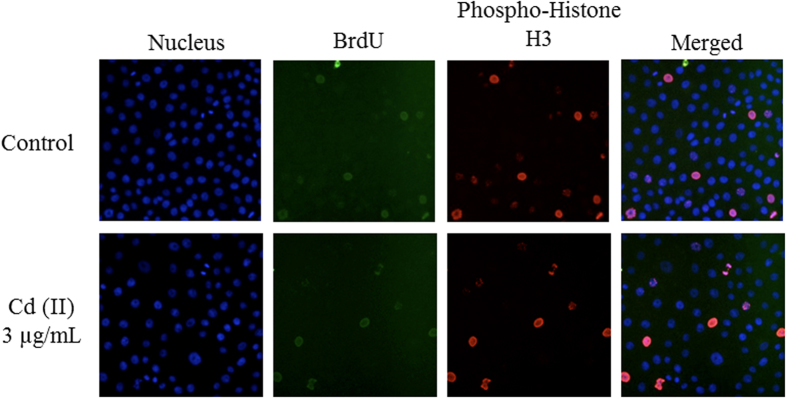# Corrigendum: Apoptotic effect of novel Schiff Based CdCl_2_(C_14_H_21_N_3_O_2_) complex is mediated via activation of the mitochondrial pathway in colon cancer cells

**DOI:** 10.1038/srep46793

**Published:** 2017-06-05

**Authors:** Maryam Hajrezaie, Mohammadjavad Paydar, Chung Yeng Looi, Soheil Zorofchian Moghadamtousi, Pouya Hassandarvish, Muhammad Saleh Salga, Hamed Karimian, Keivan Shams, Maryam Zahedifard, Nazia Abdul Majid, Hapipah Mohd Ali, Mahmood Ameen Abdulla

Scientific Reports 5: Article number: 909710.1038/srep09097; published online: 03 13 2015; updated: 06 05 2017

In this Article the images presented in Figure 2a are duplicated from Figure 4a in Reference 47. These errors are due to accidental mislabelling of data files and the figure, which resulted in an incorrect figure being mistakenly included in the paper.

The correct Figure 2a appears below as Figure 1.

## Figures and Tables

**Figure 1:**