# Supplementary material for: Apoptotic effect of novel Schiff Based CdCl2(C14H21N3O2) complex is mediated via activation of the mitochondrial pathway in colon cancer cells
Source: Sci Rep. 2015 Mar 13;5:9097. doi: 10.1038/srep09097 (PMC4649862; doi:10.1038/srep09097)
Supplement: Supplementary Information — Supplementary Table S1 corresponding assays, cell number of each assay, complex concentrations and the corresponding tables and figures. [file srep09097-s1.pdf]

Apoptotic effect of novel Schiff Based $CdCl_2(C_{14}H_{21}N_3O_2)$ complex is mediated via activation of the mitochondrial pathway in colon cancer cells

Maryam Hajrezaie[1,2], Mohammadjavad Paydar[3], Chung Yeng Looi[3], Soheil Zorofchian Moghadamtousi[2], Pouya Hassandarvish[1], Muhammad Saleh Salga[4], Hamed Karimian[3], Keivan Shams[2], Maryam Zahedifard[1,2], Nazia Abdul Majid[2], Hapipah Mohd Ali[4], Mahmood Ameen Abdulla[1]*

Supplementary table-1 corresponding assays, cell number of each assay, complex concentrations and the corresponding tables and figures.

Corresponding assays	Cell number	Corresponding complex concentration	Corresponding table/figure
Cell culture and cell viability assay	(1× 10^5 cells/mL)	0 -50 µg/mL	Table 1
LDH release assay	(1× 10^5 cells/mL)	0, 0.75, 1.5 and 3 g/ml	Figure 1
Cell cycle analysis	(1× 10^6 cells/mL)	3 µg/mL	Figure 2, 3
Acridine orange/ propidium iodide double staining	(1× 10^5 cells/mL)	3 µg/mL	Figure 4
(ROS) generation	(1× 10^5 cells/mL)	0, 0.75, 1.5 and 3 µg/ml	Figure 5
Multiple cytotoxicity assay	(1× 10^5 cells/mL)	3 µg/mL	Figure 6
Measurement of caspase activities	(1× 10^5 cells/mL)	0, 0.75, 1.5 and 3 µg/ml	Figure 7
Measurement of NF-κB activity	(1× 10^5cells/mL)	3 µg/mL	Figure 8
Western Blot experiments	(1× 10^6 cells/mL)	0, 1.5 and 3 µg/ml	Figure 9
Apoptosis Evaluation	(1× 10^5cells/mL)	3 µg/mL	Figure 10
Quantitative PCR analysis	(1× 10^6 cells/mL)	0, 1.5 and 3 µg/ml	Figure 11
